# Supplementary material for: Embryonic piRNAs target horizontally transferred vertebrate transposons in assassin bugs
Source: Front Cell Dev Biol. 2024 Nov 20;12:1481881. doi: 10.3389/fcell.2024.1481881 (PMC11614815; doi:10.3389/fcell.2024.1481881)
Supplement: Supplementary file 1 [file DataSheet1.pdf]

Supplementary Figure 1

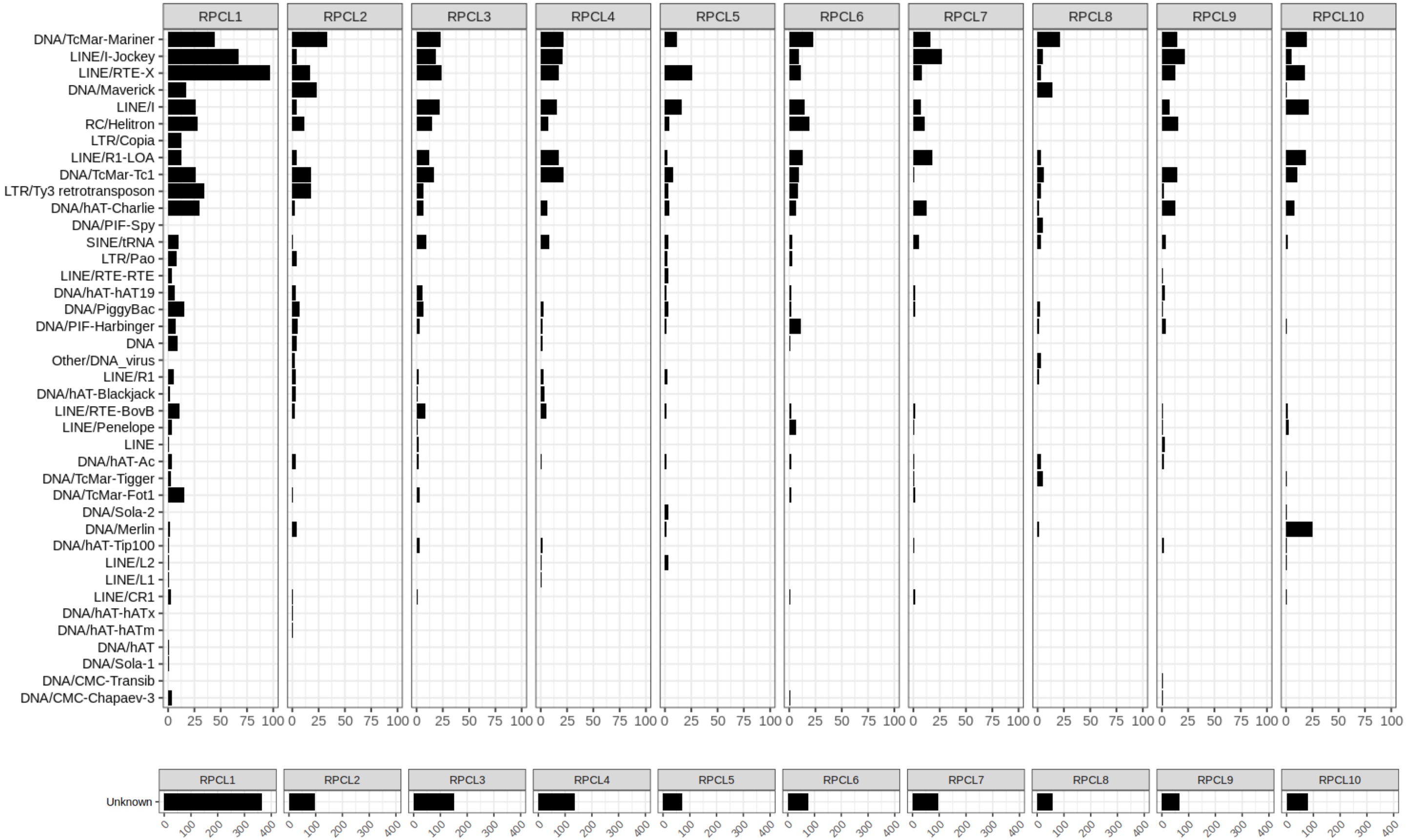

Supplementary Figure 2

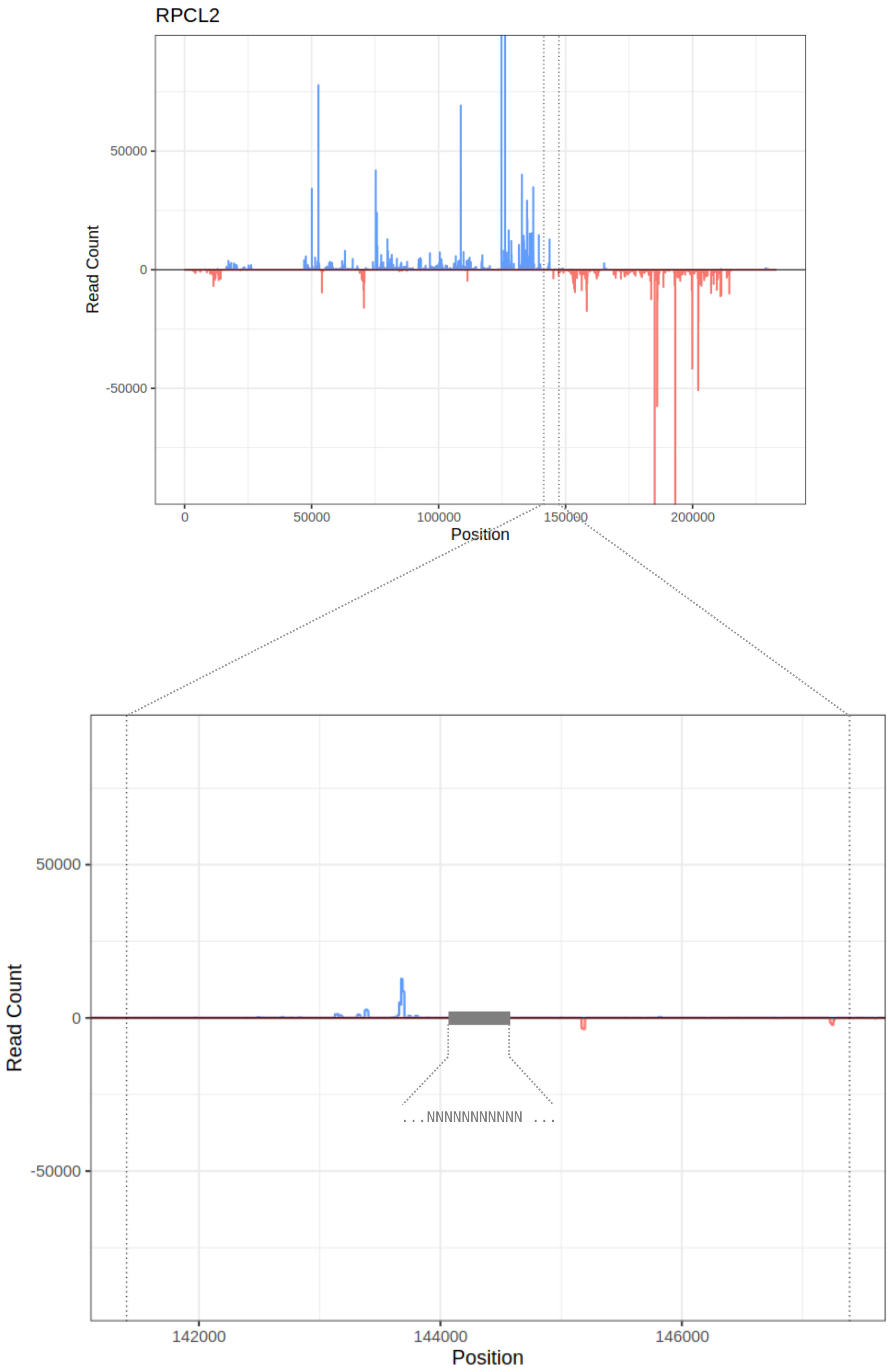

Supplementary Table 1

Sample	Total Reads	Total Reads After QC		Reads mapped to RproC3		Reads mapped to HiC assembly	
PVS1	5.582.939	4.277.071	76,61%	3.715.594	86,87%	3.715.585	86,87%
PVS2	12.367.809	9.338.953	75,51%	8.676.335	92,90%	8.676.312	92,90%
Egg1	21.504.101	10.673.452	49,63%	9.219.560	86,38%	9.219.503	86,38%
Egg2	12.581.422	10.673.452	84,84%	10.202.002	95,58%	10.201.718	95,58%
Emb1	40.816.766	36.273.048	88,87%	33.723.248	92,97%	33.721.943	92,97%
Emb2	35.811.987	32.006.382	89,37%	29.818.243	93,16%	29.817.043	93,16%
Nym1	32.934.837	30.502.999	92,62%	29.155.402	95,58%	29.155.221	95,58%
Nym2	27.271.760	25.385.570	93,08%	24.245.468	95,51%	24.245.380	95,51%

Supplementary Table 2

Sample	Number of reads associated with repeat elements	Number of piRNA reads	Number of Paired piRNA Reads	Number of Paired piRNA Reads (%)	Number of Unpaired piRNA Reads	Number of Unpaired piRNA Reads (%)
pvs1	869.438	138.187	17.284	12,51	120.903	87,49
pvs2	2.011.369	405.222	56.006	13,82	349.216	86,18
egg1	3.818.141	531.599	77.256	14,53	454.343	85,47
egg2	5.003.045	3.873.579	714.790	18,45	3.158.789	81,55
emb1	16.909.817	13.642.074	4.033.460	29,57	9.608.614	70,43
emb2	15.678.915	13.054.204	3.836.780	29,39	9.217.424	70,61
nym1	4.944.981	3.183.897	931.758	29,26	2.252.139	70,74
nym2	2.825.846	1.230.413	341.111	27,72	889.302	72,28
total	52.061.552	36.059.175	10.008.445	-	26.050.730	-
